# Comparative genomics reveals specialization and divergent virulence potential in *Vibrio vulnificus*, *Vibrio navarrensis*, and *Vibrio cidicii*

**DOI:** 10.1128/aem.01827-25

**Published:** 2025-11-18

**Authors:** Keri Ann Lydon, Megan E. J. Lott

**Affiliations:** 1 Department of Environmental Health Science, College of Public Health, University of Georgia 822554, Athens, Georgia, USA; 2 Department of Environmental Sciences and Engineering, University of North Carolina 312056, Chapel Hill, North Carolina, USA; University of Georgia Center for Food Safety, Griffin, Georgia, USA

**Keywords:** virulence potential, comparative genomics, *Vibrio cidicii*, *Vibrio navarrensis*, *Vibrio vulnificus*, One Health

## Abstract

*Vibrionaceae* are a diverse family of bacteria that contain pathogenic species, including those within the *Vulnificus* clade: *Vibrio vulnificus*, *Vibrio navarrensis*, and *Vibrio cidicii*. While *V. vulnificus* is a generally well-characterized environmental pathogen, *V. cidicii* and *V. navarrensis* are relatively rare, recently identified species that our current understanding of virulence and environmental adaptation is limited. Here, we investigate genetic relatedness across these three species to identify shared and species-specific genes, including markers of virulence. Using publicly available genome assemblies (*n* = 76), we evaluated phylogenetic and genomic diversity across this clade. We sampled all available *V. navarrensis* and *V. cidicii* genomes and a biodiverse curated set of four *V. vulnificus* ecotypes to ensure representative coverage. Our results indicate that all three species share 2,313 core genes, many of which are core bacterial functions in addition to pathways important to environmental response, host immune evasion, and iron acquisition. Moreover, *V. cidicii* and *V. navarrensis* have extensive genetic similarity between them*,* including average nucleotide identities >95% and 370 shared genes. Despite this similarity, they both remain more phylogenetically distant from *V. vulnificus* and lack key virulence genes, such as *rtxA*, indicating alternative pathogenic potential. Overall, these findings reveal distinct evolutionary strategies within the *Vulnificus* clade, with *V. vulnificus* specializing in enhanced pathogenesis, while *V. navarrensis* and *V. cidicii* have evolved enhanced environmental persistence capabilities.

**IMPORTANCE:**

*Vibrio* species are important environmental aquatic bacteria that pose a threat to human and animal health across the globe. This study applied comparative genomics to investigate the genetic relatedness of *Vibrio vulnificus*, *Vibrio navarrensis*, and *Vibrio cidicii*, with special focus on genes associated with environmental adaptation and virulence between and within each species. Results indicate *V. navarrensis* and *V. cidicii* share many genes, are phylogenetically close, and exhibit genomic signatures of enhanced environmental persistence and stress tolerance in addition to survival in anthropogenically impacted marine systems. Furthermore, *V. vulnificus* possesses an overall different virulence potential with the presence of RTX systems. This adds to our understanding of genetic diversity and pathogenic mechanisms within an important group of marine pathogens.

## INTRODUCTION

The family *Vibrionaceae* encompasses a wide range of nine genera made up of 51 clades and over 190 species, including many that are human and animal pathogens (1). In the *Vulnificus* clade, *V. vulnificus* was originally classified as the sole species (2); however, recent advancements in microbial genomics have revealed newly described and closely related *Vibrio* species (1, 3), including *Vibrio navarrensis* (4) and *Vibrio cidicii* (5).

Among these three species, *V. vulnificus* is clinically significant due to its severe pathogenic potential as one of the leading causes of death related to foodborne illness and responsible for 95% of all deaths from seafood consumption (6). Additionally, *V. vulnificus* infections have a high case fatality rate (up to 50%) and result in severe outcomes, such as necrotizing skin infections, sepsis, amputation, and death (7). Beyond foodborne illness, many infections arise from direct contact with brackish waters and marine life (8). Despite this well-characterized pathogenicity of *V. vulnificus*, the virulence potential and ecology of *V. cidicii* and *V. navarrensis* remain poorly understood, presenting a significant knowledge gap for public health risk assessment.

When compared to *V. vulnificus*, *V. navarrensis* has only recently been recognized as a human pathogen and is less frequently isolated, though it can be found in similar marine environments and sewage and has been recovered from human blood, stool, and wound samples (9–11). Beyond human illness, *V. navarrensis* has been linked to livestock (pigs and cattle) that suffered spontaneous abortions (12). In contrast, *V. cidicii* has primarily been isolated from aquatic environments and has not been classified as a human pathogen, though it has been isolated from human blood (5). Pathogenic potential across these species remains poorly characterized.

*V. cidicii* is similar to *V. navarrensis* in most phenotypical tests, except for their ability to utilize L-rhamnose as a sole carbon source (5). One recent study found *V. navarrensis* in high abundance across spatial and temporal gradients in surface waters, indicating potential for diverse environmental adaptations (13). Additionally, *V. navarrensis* has been found in biofilms on microplastics (14, 15). Despite these environmental findings, our understanding of environmental drivers of abundance for these two species remains limited.

To better understand these species relationships and pathogenic potential, this study has three primary objectives: (i) determine phylogenetic relatedness across the full spectrum of available *V. navarrensis* and *V. cidicii* genomes compared with a subset of diverse *V. vulnificus* strains; (ii) identify species-specific genetic features that may explain differences in virulence potential and environmental adaptation; and (iii) characterize the genomic diversity within each species to understand ecological and clinical strain clustering. We hypothesize that the three species within the *Vulnificus* clade represent distinct evolutionary lineages with species-specific adaptations for pathogenicity and environmental survival despite their close phylogenetic relationship. This research provides a comprehensive assessment using comparative genomics to identify the genetic basis for observed differences in virulence potential and ecological distribution.

## MATERIALS AND METHODS

### Strain selection

In this study, we obtained publicly available whole genome assemblies from the National Center for Biotechnology Information (NCBI) in August 2025 for the three species that make up the *Vulnificus* clade: *Vibrio vulnificus*, *Vibrio navarrensis*, and *Vibrio cidicii* (1). After the initial download, we discovered during curation that several of the isolates were duplicated either through using unique identifiers created by the submitter or through multiple sequencing efforts used to build complete genomes. To limit the redundancy of genome sequences, we dereplicated the isolates by strain identification numbers to include only the complete final genome for each isolate, if one was available. If the NCBI quality control was flagged for not passing quality checks, we used the available draft genome (e.g., contig-level assembly) in its place. No isolates flagged for contamination or quality issues in NCBI were included in the study.

To ensure a robust comparative analysis across species with vastly different representations in public databases, we employed a complete sampling strategy. We obtained all available genomes for the rarer species (*V. navarrensis* and *V. cidicii*) to eliminate the sampling bias while implementing curated biological diversity sampling for *V. vulnificus* to capture ecological and pathogenic diversities across established ecotypes. This approach prevents overrepresentation of *V. vulnificus* while maximizing genetic diversity representation across the clade. For *V. vulnificus*, we selected a subset of genomes (*n* = 16) evenly across previously described ecotypes C1, C2, C3, and C4 (16), making sure to include environmental and clinical isolates for each ecotype.

All genome assemblies were processed with Galaxy (17, 18) on the public server UseGalaxy.eu; unless noted, default parameters were used. We evaluated genomic features and assembly quality with QUAST v5.3.0 (19–21). High-quality genome assemblies were defined as those with N50 values > 50,000 bp and ≤301 contigs; assemblies meeting these criteria were included in subsequent analyses. In total, we obtained 76 isolates across the *Vulnificus* clade that included all isolates currently available for *V. navarrensis* (*n* = 40) and *V. cidicii* (*n* = 20). Accession numbers for genomes are listed in Table 1 with strain metadata and the original studies where the strains were previously characterized (4, 5, 10, 12, 16, 22–34).

**TABLE 1:** *Vibrio vulnificus*, *Vibrio navarrensis*, and *Vibrio cidicii* strains used in this study with the corresponding accession numbers for each assembly and the reference for each isolate’s initial appearance in the literature

Strain	Assembly accession	Species	Isolation source	Country	Collection date	Assembly level	First reference
1048-83	GCA_001597955.1	*cidicii*	Blood	USA	1983	Contig	(5)
2020RZ130	GCA_043840705.1	*cidicii*	Ocean	China	2020C	Contig	–^*c*^
2423-01^*a*^	GCA_009665415.1	*cidicii*	Blood	USA	2001	Complete	(5)
2538-88	GCA_001597935.1	*cidicii*	Blood	USA	1988	Contig	(5)
KN1	GCA_964276255.1	*cidicii*	Baltic Sea	Denmark	2023	Contig	(22)
NK1	GCA_964276245.1	*cidicii*	Baltic Sea	Denmark	2023	Contig	(22)
NK2	GCA_964276355.1	*cidicii*	Baltic Sea	Denmark	2023	Contig	(22)
NK3	GCA_964276285.1	*cidicii*	Baltic Sea	Denmark	2023	Contig	(22)
NK4	GCA_964276305.1	*cidicii*	Baltic Sea	Denmark	2023	Contig	(22)
PNUSAV002954	GCA_024491315.1	*cidicii*	Human	USA	2022	Contig	–
PNUSAV003730	GCA_033630565.1	*cidicii*	Human	USA	2022	Contig	–
RB1	GCA_964276345.1	*cidicii*	Baltic Sea	Denmark	2023	Contig	(22)
RB2	GCA_964276275.1	*cidicii*	Baltic Sea	Denmark	2023	Contig	(22)
RB3	GCA_964276335.1	*cidicii*	Baltic Sea	Denmark	2023	Contig	(22)
SB1	GCA_964276265.1	*cidicii*	Baltic Sea	Denmark	2023	Contig	(22)
SB2	GCA_964276295.1	*cidicii*	Baltic Sea	Denmark	2023	Contig	(22)
SK1	GCA_964276325.1	*cidicii*	Baltic Sea	Denmark	2023	Contig	(22)
VC01	GCA_051352415.1	*cidicii*	Seawater	China	2019	Complete	–
VN-3125	GCA_015709065.1	*cidicii*	Seawater	Sweden	2011	Contig	(12)
VN-3139	GCA_015709045.1	*cidicii*	Seawater	Sweden	2011	Contig	(12)
0053-83^*a*^	GCA_012275065.1	*navarrensis*	Wound	USA	1983	Complete	(10)
0706Y	GCA_040530935.1	*navarrensis*	Raw squid	Thailand	2023	Contig	–
08-2462	GCA_009665215.1	*navarrensis*	Blood	USA	2008	Complete	(10)
20-VB00237	GCA_015767675.1	*navarrensis* ^*b*^	Wound	Germany	2020	Complete	–
2014 V-1106	GCA_012274885.1	*navarrensis*	Unknown	USA	2014	Complete	–
2021 V-1020	GCA_024216375.1	*navarrensis*	Human	USA	2021	Contig	–
2021 V-1097	GCA_024216475.1	*navarrensis*	Human	USA	2021	Contig	–
2021 V-1098	GCA_024217415.1	*navarrensis*	Human	USA	2021	Contig	–
2023 V-1015	GCA_036968175.1	*navarrensis*	Human	USA	2023	Contig	–
2023 V-1173	GCA_037161605.1	*navarrensis*	Human	USA	2023	Contig	–
2023 V-1219	GCA_039890895.1	*navarrensis*	Blood	USA	2023	Contig	–
2232	GCA_000764355.1	*navarrensis*	Sewage	Spain	1982	Contig	(4)
2462-79	GCA_009763725.1	*navarrensis*	Wound	USA	1979	Complete	(10)
ATCC-51183 (2540-90)	GCA_000764325.1	*navarrensis*	Sewage	Spain	1982	Complete	(4)
DA9	GCA_016611225.1	*navarrensis*	Seawater	USA	2014	Contig	(23)
PNUSAV000199	GCA_015803995.1	*navarrensis*	Unknown	USA	unknown	Contig	–
PNUSAV002826	GCA_024103755.1	*navarrensis*	Human	USA	2021	Contig	–
PNUSAV003264	GCA_025858195.1	*navarrensis*	Human	USA	2022	Contig	–
PNUSAV004886	GCA_032616655.1	*navarrensis*	Human	USA	2023	Contig	–
PNUSAV005410	GCA_051366045.1	*navarrensis*	Human	USA	2025	Contig	–
PNUSAV006652	GCA_048162665.1	*navarrensis*	Wound	USA	2024	Contig	–
VN-0392	GCA_014925455.1	*navarrensis*	Cattle placenta	Germany	1999	Contig	(12)
VN-0413	GCA_014905665.1	*navarrensis*	Cattle placenta	Germany	2000	Contig	(12)
VN-0414	GCA_014925475.1	*navarrensis*	Cattle placenta	Germany	2000	Contig	(12)
VN-0415	GCA_014925505.1	*navarrensis*	Cattle fetus	Germany	2009	Contig	(12)
VN-0506	GCA_014925465.1	*navarrensis*	Cattle placenta	Germany	2000	Contig	(12)
VN-0507	GCA_014925445.1	*navarrensis*	Cattle placenta	Germany	2000	Contig	(12)
VN-0508	GCA_014925545.1	*navarrensis*	Pig placenta	Germany	2000	Contig	(12)
VN-0509	GCA_014925575.1	*navarrensis*	Pig fetus	Germany	2001	Contig	(12)
VN-0510	GCA_014925675.1	*navarrensis*	Unknown	Germany	unknown	Contig	(24)
VN-0511	GCA_014905675.1	*navarrensis* ^*b*^	Seawater	Germany	unknown	Contig	(24)
VN-0512	GCA_014905715.1	*navarrensis* ^*b*^	Seawater	Germany	unknown	Contig	(24)
VN-0513	GCA_014905775.1	*navarrensis* ^*b*^	Seawater	Germany	unknown	Contig	(24)
VN-0514	GCA_014925555.1	*navarrensis*	Pig placenta	Germany	2007	Contig	(12)
VN-0515	GCA_014925585.1	*navarrensis*	Pig placenta	Germany	2007	Contig	(12)
VN-0516	GCA_014925595.1	*navarrensis* ^*b*^	Brackish water	Germany	2015	Contig	(12)
VN-0517	GCA_014905705.1	*navarrensis* ^*b*^	Seawater	Germany	2015	Contig	(12)
VN-0518	GCA_014925645.1	*navarrensis* ^*b*^	seawater	Germany	2015	Contig	(12)
VN-0519	GCA_014925665.1	*navarrensis*	Blue mussel	Germany	2011	Contig	(12)
WAPHLVBRA00023	GCA_016094345.1	*navarrensis*	Swab	USA	2017	Contig	–
12	GCA_002074885.2	*vulnificus*	Unknown	Israel	1996	Contig	(25)
32	GCA_002903885.1	*vulnificus*	Blood	Israel	1996	Contig	(26)
93U204	GCA_000746665.1	*vulnificus*	Tilapia	Taiwan	2004	Complete	(27)
94385	GCA_002903695.1	*vulnificus*	Wound	Spain	2001	Contig	(26)
99-796 DP-E7	GCA_001890485.1	*vulnificus*	Oyster	USA	1999	Contig	(28)
ATCC 27562 (NBRC 15645)^*a*^	GCA_002224265.1	*vulnificus*	Blood	USA	1979	Complete	(29)
ATCC 29306 (CDC A1402)	GCA_001471415.2	*vulnificus*	Eye ulcer	USA	unknown	Contig	(30)
ATCC 29307 (CDC A8694)	GCA_001471465.2	*vulnificus*	Blood	USA	unknown	Contig	(30)
ATCC-43382 (VVL1)	GCA_001471305.2	*vulnificus*	Blood	USA	unknown	Contig	(31)
FORC_009	GCA_001433435.1	*vulnificus*	Stool	South Korea	2008	Complete	(26)
VN-0092	GCA_002073185.1	*vulnificus*	Blood	Germany	2011	Contig	(32)
VN-0104	GCA_002073105.1	*vulnificus*	Seawater	Germany	2010	Contig	(32)
VN-0112	GCA_002073145.1	*vulnificus*	Brackish water	Germany	2010	Contig	(32)
VV4-03	GCA_000743095.1	*vulnificus*	Wound	Israel	2003	Contig	(33)
VVyb1-BT3	GCA_000342305.2	*vulnificus*	Tilapia	Israel	2004	Complete	(34)
WAPHLVBRA00001	GCA_002278495.1	*vulnificus*	Tilapia	USA	2016	Contig	(16)

^*a*^ Reference Strain.

^*b*^ Biotype *pommerensis.*

^*c*^ “–” indicates either an unfound initial reference or this is the first time the genome is appearing in the literature.

### Genome analyses

We used Prokka v1.14.6 (35, 36) to annotate genomes for downstream analyses. To identify conserved orthologous genes across the *Vulnificus* clade, we processed all genome annotations with ROARY v3.13.0 (37). ROARY was specifically chosen for its conservative gene clustering approach, which has been successfully used for interspecies comparative analyses within bacterial genera (38). We adjusted ROARY’s default parameters to ensure better detection of orthologous groups for interspecies comparisons by reducing the identity threshold for BlastP to 70% (39) and MCL inflation to 1.4 to reduce the number of false positives. Core genes were defined as those present in all three species (99% of isolates), and we used the -s filter to avoid splitting paralogous gene clusters based on conserved neighborhood analysis, focusing on functional gene presence rather than copy number variation. The full pan-genome is visualized in Fig. S1.

We used IQ-TREE v2.4.0 (40) with ModelFinder (41) and 1,000 ultrafast bootstraps (42) to infer the best-scoring maximum likelihood tree from the core gene alignment of core genes produced with ROARY. We rooted the tree at the midpoint and visualized it with iTOL v7 (43). The average nucleotide identity (ANI) was estimated using fastANI v1.3 (44), then visualized as a heatmap with R v. 4.5.0, Rstudio (2025.05.1+513), using packages ‘ggdendro’ and ‘ggplot.’ The heatmap was clustered by inferring a dendrogram based on the hierarchical clustering (45) of the gene presence-absence table produced by ROARY.

To determine which genes were significantly associated with each species and isolation sources (clinical, water, aquatic animal, livestock), we used SCOARY v1.6.16 (46). Briefly, each species or category was depicted as a discrete binomial trait (i.e., belonging to the species or not). Species-specific genes were reported if they had a *P*-value < 0.05 for naive-p, Bonferroni, and Benjamini-Hochberg tests, and specificity and sensitivity were ≥95%. Isolation-specific genes were reported if *P*-value < 0.05 for naive-p, Bonferroni, and Benjamini-Hochberg tests; sensitivity and specificity were reported with each significant gene.

For functional classification, the core gene alignment was processed into a single consensus sequence using the ‘Consensus sequence from aligned FASTA’ tool v.1.0.0 (47), with the most frequent nucleotide as the consensus model. The consensus sequence was then processed with eggNOG v. 2.1.8 (48, 49) to determine the Kyoto Encyclopedia of Genes and Genomes (KEGG) orthology (50) and gene ontology (GO) categories (51). Clusters of Orthologous Groups (COG) categories for core genes were visualized in R (52).

### Virulence factors and antimicrobial resistance

We screened each strain individually with ABRicate v1.0.1 (53) for the presence of virulence factors using the Virulence Factors of Pathogenic Bacteria (VFDB) (54) database and antimicrobial resistance genes against the Comprehensive Antibiotic Resistance Database (CARD) (55), with 70% minimum nucleotide identity and coverage.

## RESULTS

### Genomic features

In this study, we evaluated the relationship between species within the *Vulnificus* clade, including *V. vulnificus*, *V. navarrensis*, and *V. cidicii*. Assembly quality metrics (e.g., contig counts, N50 values, and total assembly lengths) are provided in Table S1. Examination of genomic features indicated that *Vulnificus* clade genomes (*n* = 76) were on average 4,705,000 bp (range 4,139,000–5,744,000 bp), with a mean GC content of 47.8% (range 46.4–48.6%). When looking at each species individually, *V. vulnificus* (*n* = 16) had the largest mean genome size of 5,059,000 bp (range 4,680,000–5,745,000 bp), while *V. navarrensis* (*n* = 40) and *V. cidicii* (*n* = 20) were 4,564,000 bp (range 4,139,000–5,110,000 bp) and 4,628,000 bp (range 4,389,000–5,048,000 bp), respectively. Mean GC content was 46.7% (range 46.4–47.9%) for *V. vulnificus* isolates and 48.3% (range 47.7–48.6%) and 48.0% (range 46.7–48.3%) each for *V. navarrensis* and *V. cidicii*, respectively.

### Phylogenetic analysis

**Fig 1:**
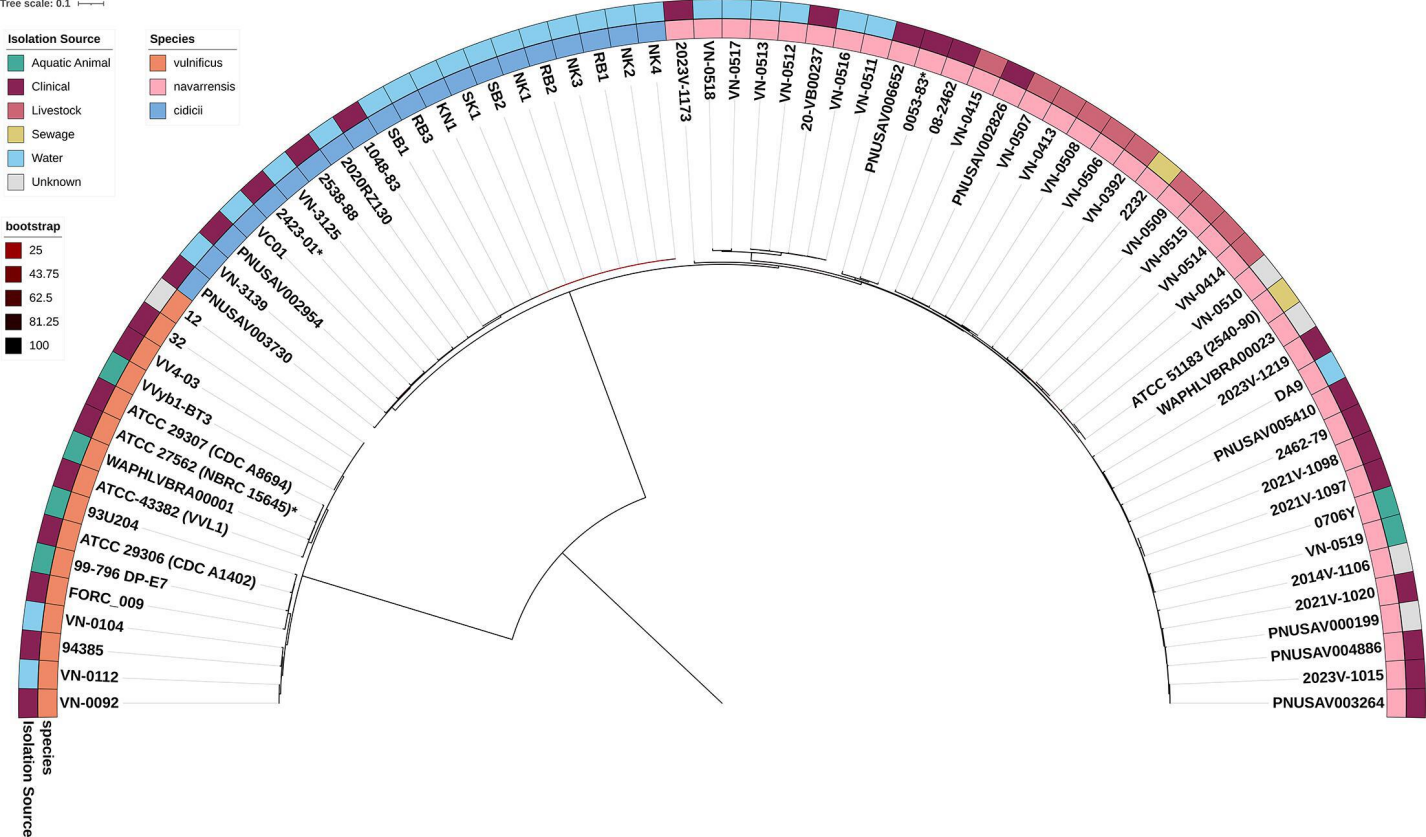
Phylogenetic tree inferred from the concatenated alignment of core genes for all three species of the *Vulnificus* clade: *Vibrio vulnificus*, *Vibrio navarrensis*, and *Vibrio cidicii*. Maximum likelihood phylogeny was constructed using IQ-TREE v2.4.0 with ModelFinder (GTR + F + I + R6 model) and 1,000 ultrafast bootstraps from 1,449,302 nucleotide sites. The tree is midpoint-rooted. Strain names and isolation sources are indicated for each isolate.

ModelFinder determined the best-fit model for maximum likelihood phylogeny reconstruction across 1,449,302 nucleotide sites from core genes to be GTR + F + I + R6 (general time reversible with empirical base frequencies, invariant sites, and six-category rate heterogeneity). The resulting phylogenetic tree revealed clear interspecies relationships with well-supported clades (Fig. 1). The tree exhibited a clear bifurcating structure with *V. vulnificus* forming one monophyletic clade, with all four ecotype clusters resolved, and *V. navarrensis* and *V. cidicii* forming a separate sister clade. All species-level nodes achieved 100% bootstrap support values.

*V. navarrensis* displayed high intraspecies genetic diversity, forming multiple phylogenetic subclades with bootstrap values ranging from 47 to 100%. Lower bootstrap values were concentrated at nodes within the *V. navarrensis* clade, particularly among environmental and livestock-associated lineages. Livestock-associated isolates from cattle and pig spontaneous abortion cases showed phylogenetic clustering. In contrast, environmental isolates from seawater, sewage, and brackish water were phylogenetically dispersed throughout the tree. Clinical isolates from blood, wound, stool, and unspecified clinical sources were distributed across multiple phylogenetic lineages. Of particular interest, a recent clinical isolate (PNUSAV002826, isolated in 2021) clustered with livestock isolates from cattle fetal and placental tissues. Similarly, the environmental isolate *V. navarrensis* DA9 from a coastal canal in the Florida Keys (USA) clustered closely with recent USA clinical isolates 2021V-1098, 2021V-1097, and 2023V-1219.

**Fig 2:**
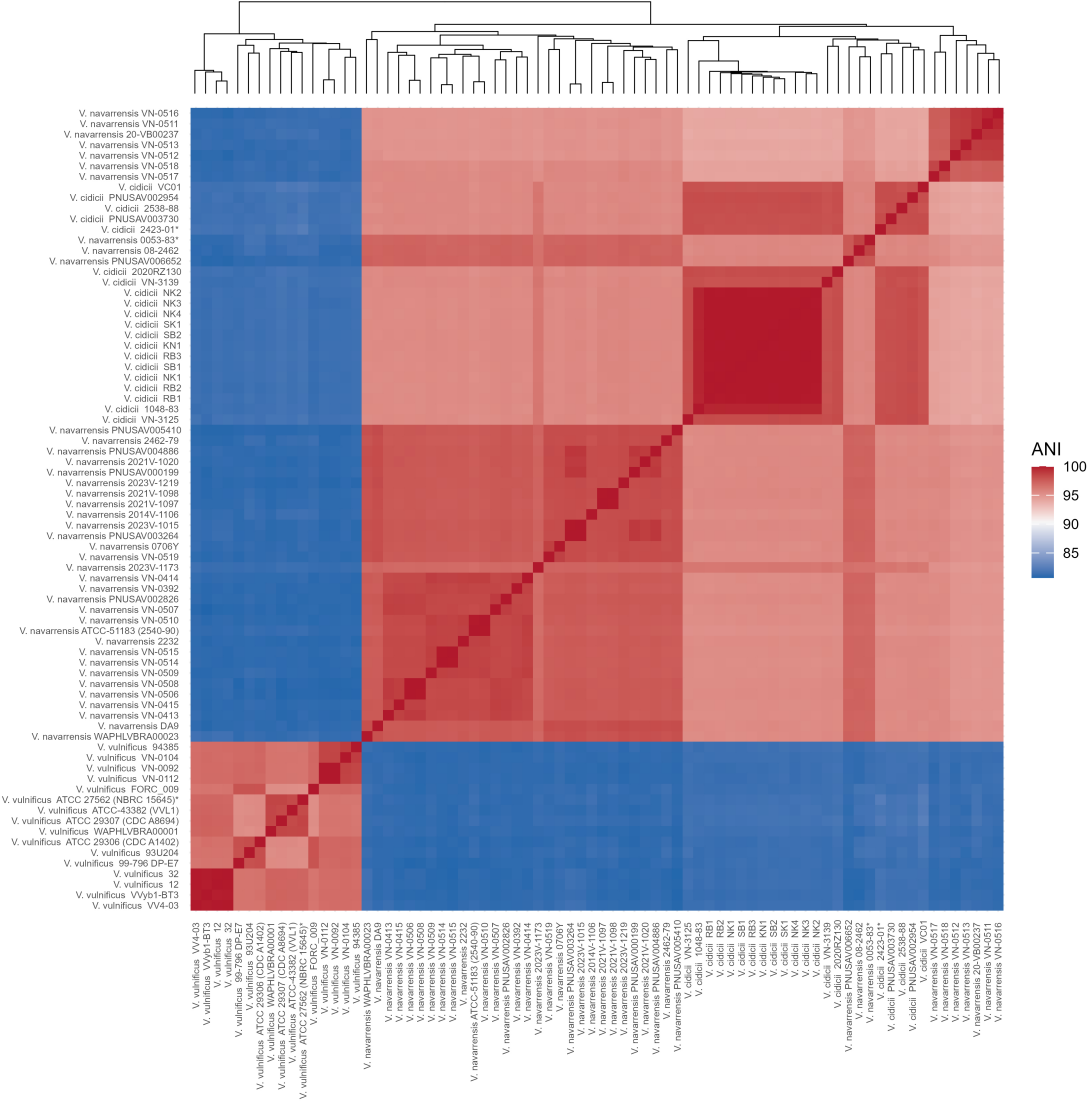
Pairwise average nucleotide identity (ANI) across the *Vulnificus* clade. The ANI values were calculated using fastANI v1.3 and visualized as a heatmap with hierarchical clustering. The dendrogram indicates clustering based on the gene presence-absence profiles produced by ROARY at a 70% identity threshold.

In contrast, hierarchical clustering based on gene presence/absence at 70% amino acid identity threshold showed clusters of *V. navarrensis* and *V. cidicii* isolates intermixed (Fig. 2), contrasting with the clear phylogenetic separation observed in the core genome phylogenetic tree.

### Average nucleotide identity (ANI) analysis

Across the *Vulnificus* clade, the average nucleotide identity (ANI) was on average 90.8% with a range of 80.7 to 100% (Fig. 2). Intraspecies comparisons revealed higher ANI values, as expected. We observed average intraspecies ANI values of 98.8 (SD = 0.9), 97.3 (SD = 1.3), and 97.2% (SD = 1.3) for *V. cidicii*, *V. navarrensis*, and *V. vulnificus*, respectively. Interspecies pairwise comparisons identified distinct patterns; both *V. cidicii* and *V. navarrensis* had lower ANI values when compared to *V. vulnificus* (81.5% ± 0.2% and 81.1% ± 0.7%, respectively). Contrarily, the pairwise comparison between *V. navarrensis* and *V. cidicii* had an average ANI value of 95.4% (SD = 0.7). Within *V. navarrensis*, biotype *pommerensis* strains showed almost identical average ANI values of 95.5% (SD = 1.0) when compared to all non-pommerensis *V. navarrensis* isolates. *V. navarrensis* biotype *pommerensis* strains among themselves had average ANI values of 98.5% (SD = 0.98).

### Core genes

**Fig 3:**
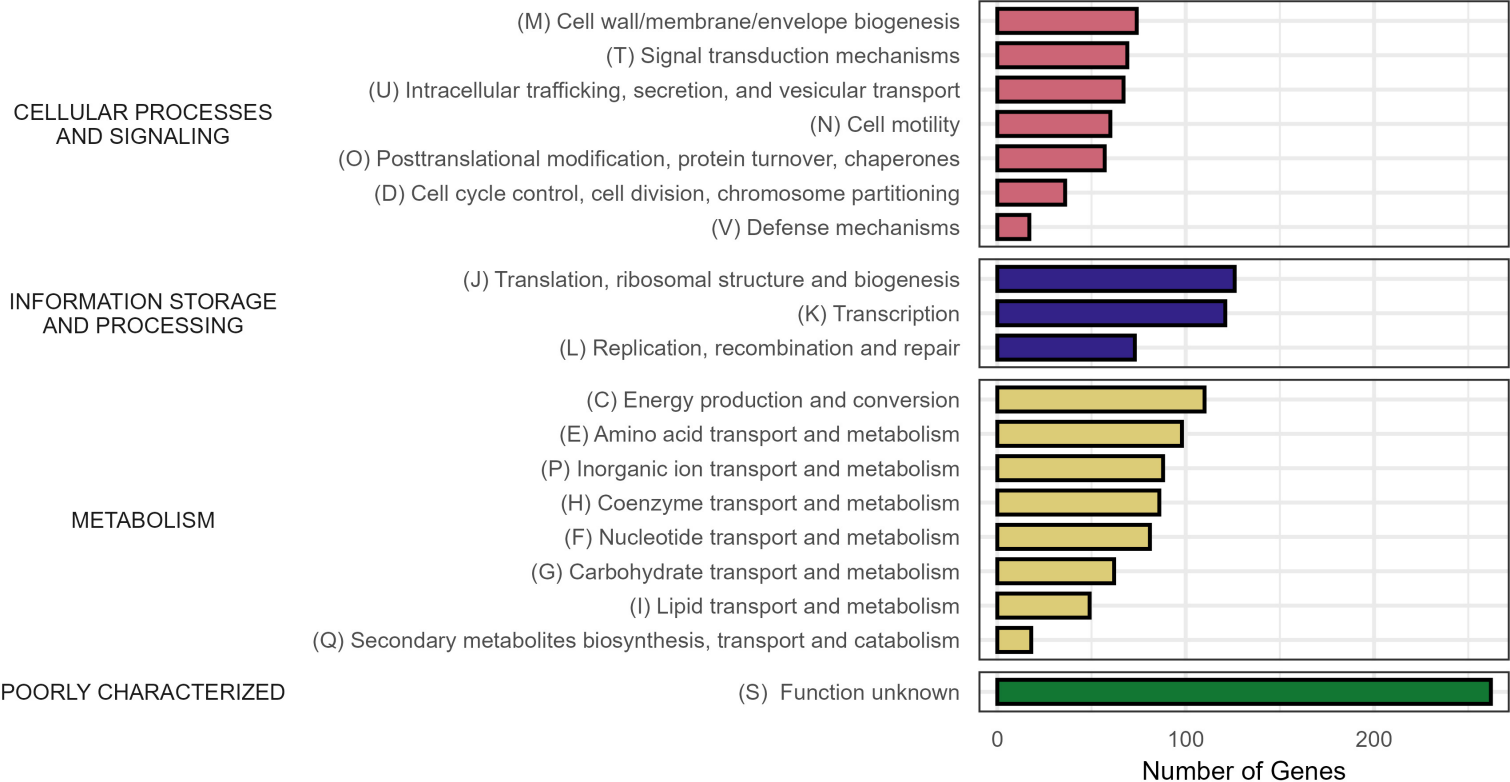
Functional categorization of the core conserved genes across the *Vulnificus* clade. Of the 2,313 core conserved genes (present in 99–100% of strains), 1,459 were functionally annotated using eggNOG-mapper v2.1.8 and assigned to the Clusters of Orthologous Groups (COG) categories. The bars indicate the numbers of genes assigned to the COG categories.

We identified 2,313 core genes (99–100% of strains) across all three species. An additional 188 highly conserved genes (95–99% of strains), 3,038 shell genes (15–95% of strains), and 9,861 cloud genes (0–15% of strains) were found across all isolates. Functional annotation of core genes with eggNOG-mapper assigned COG categories to 1,459 genes, representing all major COG functional categories (Fig. 3). Metabolism-related functions were the largest category (38.1%), followed by cellular processes and signaling (24.5%) and information storage and processing (20.6%). The largest singular COG categories were those that were poorly characterized with unknown functions (S). This was followed by core bacterial processes of translation, ribosomal structure and biogenesis (J), transcription (K), and energy production and conversion (C). Interestingly, there were some conserved functions across inorganic ion transport and metabolism (P).

We identified many pathogenicity-associated genetic elements conserved across all three species. Complete flagellar biosynthesis and motility systems were present, including structural (*fliG*, *fliM*, *fliN, flgB*, *flgC*, *flgF*, *flgH*, *flgI*), regulatory (*fliAS*), and chemotactic components (*cheA, cheY*, *cheZ*), alongside Type IV pilus assembly machinery (*pilA*, *pilT*, *pilQ*). Intracellular communication capabilities included quorum sensing regulatory circuits (*luxS*, *luxR*, *luxT*) and two-component regulatory systems (*cpxA*, *cpxR*, *phoR*). Environmental adaptation mechanisms were equally well-conserved, including oxidative stress defense systems (*katG*, *sodB*, *oxyR*), iron acquisition pathways (*hmuT*, *hmuU*, *hmuV*, *feoA*, *feoB*, *bfr, fur*, *yclN*, *yclO*, *yclP*, *yclQ*), histidine utilization pathways (*hutG*, *hutH*, *hutI*, *hutX*, *hutZ*), and heavy metal resistance genes (*copA*, *zntA*, *znuB*, *znuC*, *zur*).

ABRicate screening revealed distinct antimicrobial resistance and virulence factor profiles. Core genes associated with resistance included the cAMP receptor protein gene (*CRP*) and tetracycline resistance gene*, tet* (*35*), present in all 76 strains. VFDB analysis identified core virulence factors across the clade, including immunolipoprotein A (*ilpA*), outer membrane protein U (*ompU*), type II secretion system pseudopilin (*gspG*), catalase (*katB*), KDO synthase (*kdsA*), quorum sensing enzyme (*luxS*), and thermolabile hemolysin (*tlh*) that were present across all three species. While not present in every strain, *ugd* was present in 22 *V*. *navarrensis* strains, five *V*. *cidicii* strains, and two *V*. *vulnificus* strains.

### Genes shared between *V. navarrensis* and *V. cidicii*

We identified genes significantly associated with each species, biotype, and shared between *V. navarrensis and V. cidicii* with SCOARY (Table S2). We identified 370 genes shared between *V. cidicii* and *V. navarrensis*, of which 208 were hypothetical proteins. The 162 annotated shared genes represented a broad range of processes for environmental persistence and pathogenicity. These included genes for ectoine biosynthesis (*ectA*, *ectB*, *ectC*), osmotic stress transport (*ousW*, *ousX*, *proV*, *gdx*), UV DNA repair systems (*phrA*, *phrB*, *alkA*), cytochrome bo3 oxidase complex (*cyoA*, *cyoB*, *cyoC*, *cyoD*, *cyoE*), cytochrome bd-II oxidase (*appB*, *appC*), sugar metabolism (*treP*, *scrB*, *scrK*, *bglA*), benzoate degradation (*benM*), and a blue light and temperature-regulated antirepressor (*bluF*). Moreover, *V. cidicii* and *V. navarrensis* share important pathogenic associated genes *cqsA* (CAI-1 quorum sensing), *comR* (competence/biofilm repressor), *csgD* (curli fimbriae regulator), and *ptrB* (protease 2).

ABRicate screening with VFDB identified *lpxC* present in all strains but absent from *V. vulnificus*. Additionally, *fcl* and *gmd* were present only in *V. navarrensis* and *V. cidicii* strains; however, they were only sporadically positive across isolates. No antimicrobial resistance genes were shared between these two species.

### *Vibrio navarrensis* unique genes

For the species *V. navarrensis*, there were no genes that met the criteria of all three *P*-values less than 0.05 in addition to sensitivity and specificity greater than or equal to 95%. However, when examining *V. navarrensis* biotype *pommerensis* individually, we found that there were 90 genes that were associated with the biotype, of which 62 were hypothetical proteins. The annotated genes included those for an RND family efflux pump (*acrF*), fucose (*fucA*), and a toluene efflux exporter (*ttgA*).

Results from VFDB screening indicated that *katA* was present in 17 *V*. *navarrensis* strains and not present in other species. Additional genes included one *V*. *navarrensis* strain positive for *ddhA* and another for *sul2*.

### *V. cidicii* unique genes

For *V. cidicii* strains, 23 genes were unique to the species, of which six were hypothetical proteins. *V. cidicii* showed metabolic specialization among its 17 annotated species-specific genes, particularly rhamnose catabolism (*rhaA*, *rhaB*, *rhaD*, *rhaM*, *rhaT*) and alcohol metabolism (*adhB_2*), as well as tetrathionate respiration capability (*ttrR*, *ttrS*). We also observed a complete paraquat-inducible (PQI) pathway (*pqiA*, *pqiB*, *pqiC*).

### *V. vulnificus* unique genes

For *V. vulnificus*, there were 796 genes unique to the species, including 417 hypothetical proteins. Among the 379 annotated genes, environmental response genes for chemotaxis (*cheA*, *cheB*, *cheD*, *cheR*, *cheW*), superoxide dismutase (*sodA*, *sodC*), chitin metabolism (*deaA*, *gbpA*), oxygen sensing (*dosP*), and oxidative damage protection (*npr*). Moreover, specific pathogenicity genes included hemolysin secretion (*hlyB*, *hlyD*), host invasion and adherence (*cadA*, *cadB*, *cadC*), leukotoxin activator (*ltxC*), and RTX toxin (*rtxA*). We observed additional genes unique to *V. vulnificus* that included siderophores vibriobactin (*vibB, viuB*) and mycobactin (*mbtA*, *mbtB*) and a ferric aerobactin receptor (*iutA*) in addition to various genes involved in iron processes: iron export (*fetB*), iron acquisition (*pchB*), and hydroxamate transport to scavenge iron from hosts (*fhuA*, *fhuB*, *fhuC*, *fhuD*).

Similarly, we observed virulence factors unique to *V. vulnificus* by screening against the VFDB database, including *varG*, *rtxB*, *rtxC*, *rtxD*, and *gmhA/lpcA*. CARD screening indicated *catB9* present in 15/16 *V*. *vulnificus* strains but was also present in one of 20 and two of 40 *V*. *navarrensis* strains. One *V. vulnificus* strain (99-796 DP-E7) was positive for T6SS genes *VCA0109*, *clpB/vasG*, *hcp-2*, *icmF/vasK*, *vasA*, *vasB*, *vasC*, *vasD*, *vasE*, *vasF*, *vasH*, *vasJ*, *vasL*, *vipA/mglA*, and *vipB/mglB*.

### Isolation source-associated genes

We found no significant genes with high specificity or sensitivity associated with isolates from aquatic animals nor clinical samples when these traits were screened with SCOARY. However, livestock and water isolation sources were significantly associated with some genes with varying sensitivity and specificity. For strains isolated from water, there were 52 significant genes of which 43 were annotated as hypothetical proteins. Of those that were identified, those with 100% specificity included *vspR*, *capV*, *dncV*, and *rnlA/B*. For livestock strains, we identified seven significant genes with variable specificity and sensitivity; these included *ytfE* (100% sensitivity, 86.8% specificity), *gdh* (100% sensitivity, 91.2% specificity), *ybdG* (100% sensitivity, 91.2% specificity), and *hin* (66.7% sensitivity, 100% specificity).

## DISCUSSION

### Phylogenetic relationships

Our interspecies comparison across the *Vulnificus* clade provides insights into evolutionary relationships and functional divergence within this clinically important *Vibrionaceae* clade. We employed complete sampling of all available genomes for the rare species *V. navarrensis* and *V. cidicii* and biologically diverse sampling for the well-characterized pathogen *V. vulnificus*. All phylogenetic relationships compare to those in previous work (1, 22); however, there has been continued emphasis on a close relationship between *V. navarrensis* and *V. cidicii* to *V. vulnificus* (5, 10) without defining features that characterize those relationships. Results presented here help clarify some of those features, including virulence potential, environmental specialization, and metabolic capabilities.

Many of the genes conserved across all three species represent core bacterial functions, though many remain uncharacterized. Of the shared pathways that are characterized, many involve inorganic ion transport systems. The conservation of these extensive inorganic ion transport systems in the core genome is particularly noteworthy, while basic iron transport systems, such as *feoA*, *feoB*, and *feoC*, are commonly conserved across bacterial species (56), the conservation of diverse metal transport systems (zinc and copper) in the core genome is not. This pattern in the *Vulnificus* clade suggests dual environmental-host survival strategies allowing persistence across diverse metal bioavailability conditions where these metals serve as both essential nutrients and potential toxins and within hosts where these systems support survival during infection (57).

The relationship between *V. navarrensis* and *V. cidicii* reveals additional complexity. These two species share a substantial number of genes (*n* = 370) that are not present in *V. vulnificus*. These findings suggest a more complex evolutionary history, including a likely recent common ancestor and a subsequent divergence of *V. cidicii* and *V. navarrensis*. This evolutionary dynamic is common in aquatic bacteria and may reflect adaptation to overlapping but distinct ecological niches (58). Mixed clustering patterns emerge when comparing gene presence/absence versus core genome phylogenetic evidence, indicating possible recombination and/or horizontal gene transfer across the accessory genomes of both species. Moreover, the average ANI values between *V. navarrensis* and *V. cidicii* (95.4%) are comparable to those between *V. navarrensis* biotype *pommerensis* and non-pommerensis *V. navarrensis* (95.5%), both falling within a critical range sometimes used for species delineation (44, 59, 60).

We have identified multiple lines of evidence of a close genetic relationship between *V. cidicii* and *V. navarrensis*, including (i) close phylogenetic relationships, (ii) similar ecological adaptations, (iii) extensive shared core and accessory genes, and (iv) ANI values marginally above the 95% threshold. While ANI values above 95% can support same species designation, additional evidence is needed for robust taxonomic conclusions. The limited availability of *V. cidicii* genomes with several closely related isolates from similar geographic regions constrains the strength of any taxonomic proposal at this time. Based on the available evidence, we hypothesize *V. cidicii* may represent another biotype of *V. navarrensis*; however, this hypothesis requires continued investigation as additional genomes become available. Future re-evaluation using multiple approaches, including synteny analysis, would provide stronger evidence for assessing their taxonomic status.

### Ecological and evolutionary implications

*Vibrio* spp. are ubiquitous aquatic bacteria, with a wide distribution across brackish and marine waters. While the ecology of *V. vulnificus* in marine systems is well-described, much less is known about the ecology and habitat range of *V. navarrensis* and *V. cidicii*. Our genomic findings, paired with previous environmental observations, suggest that these two species are broad environmental generalists that can persist across varied aquatic environments (i.e., fresh, brackish, and marine waters).

Globally, *V. navarrensis* has been identified in aquatic environments, such as the Baltic Sea (12, 24), coastal areas near Hong Kong (15), the coastal Florida Keys (23, 61), and from brackish water (12, 24, 62). Unlike *V. vulnificus*, *V. navarrensis* can grow at low salinities and without NaCl (4), indicating that exposures expand to include freshwater. *V. navarrensis* has been found in high abundance across the year in coastal areas where salinities ranged from 4.6 to 33.5 ppt (13). Conversely, *V. vulnificus* thrives from 5 to 25 ppt salinity and is most often associated with brackish waters (63). The expanded salinity range of *V. navarrensis* has implications for public health, as this species appears to thrive across fresh, brackish, and marine waters in estuarine systems where tidal mixing creates salinity gradients. Limited sampling within the narrow salinity range typical of *V. vulnificus* may miss *V. navarrensis* populations. This may explain why this species is rarely isolated despite its apparent broad distribution.

The genomic evidence in this study corroborates the assertion that *V. cidicii* and *V. navarrensis* are environmental generalists. The genes shared between *V. cidicii* and *V. navarrensis* allow for environmental persistence across a wide range of salinities, dissolved oxygen, and anthropogenic influence. For example, we identified ectoine biosynthesis (*ectA*, *ectB*, *ectC*) exclusive to these two species, which would allow for increased halotolerance (64). Moreover, we identified two pathways associated with dissolved oxygen, cytochrome bo3 oxidase complex for high oxygen conditions, such as those in shallow and fast-moving waters, and cytochrome bd-II oxidase for low oxygen environments, such as eutrophic waters. Additional shared genes include environmental stress response pathways, such as osmotic stress transport, UV DNA repair, and benzoate degradation.

Interestingly, these two species were positive for a gene with significant amino acid similarity to *bluF*, a blue light low-temperature regulator antirepressor (65). In other species, such as *Escherichia coli*, this gene is associated with environmental response to blue light and low temperatures where biofilm production is regulated (66). While this has been documented in other aquatic bacterial species (67), this is a unique finding within *Vibrio* where, to our current knowledge, *bluF* has not been documented before.

With very few isolates in existence, characterizing environmental patterns of *V. cidicii* reservoirs is limited to the known locations of isolation, such as the Baltic Sea (22). However, we can make some inferences on potential environmental adaptations from genetic evidence in this study. The combination of rhamnose catabolic genes and tetrathionate respiration genes as species-specific features of *V. cidicii* indicates they have metabolic capabilities for specialization in environments where rhamnose is present, such as algal exudates, bacterial exopolysaccharides, and dissolved organic matter (DOM) (68, 69). Our results are supported by previous characterizations of *V. cidicii* being able to use rhamnose as a sole carbon source (5). This contrasts with both *V. vulnificus* and *V. navarrensis*, who did not share that characteristic. Moreover, *V. cidicii* genomes contained paraquat-inducible (PQI) genes, which allow for survival under oxidative stress and exposure to paraquat, a widely used bipyridinium herbicide (70). For *V. cidicii*, this suggests pathogenic potential. In other bacteria, rhamnose utilization is associated with lipopolysaccharide and biofilm production (71); tetrathionate respiration provides competitive advantage during inflammation (72); and PQI systems confer oxidative stress resistance (70). These genes are present in all *V. cidicii* genomes screened, including both environmental and clinical strains, suggesting pathogenic capabilities are present prior to host transmission.

The presence of aquatic environments with algal presence, in addition to genes involved in oxidative stress response, suggests potential adaptation to eutrophic environments where *V. vulnificus* has been noted to follow algal blooms during eutrophication (73). If *V. cidicii* represents a closely related lineage or biotype of *V. navarrensis*, it is possible that it is evolving through environmental anthropogenic pressures to produce an ecotype that is in the process of speciation through environmental adaptation (13). Similarly, *V. navarrensis* biotype *pommerensis* appears to have potential for anthropogenic pressures on its evolutionary trajectory with the presence of toluene efflux pumps. Toluene is an aromatic hydrocarbon compound that has been documented within the Baltic Sea environments (74), from which many *V. navarrensis* strains have been isolated.

### Virulence mechanisms and antimicrobial resistance

Our comparative genome analysis identified several virulence-associated genes that are conserved across the *Vulnificus* clade, including iron acquisition systems and outer membrane proteins, which are often associated with host adaptations and immune evasion. Furthermore, all species in the clade were positive for hemolysin genes (*tlh*). In *V. vulnificus*, the *tlh* is species-specific and known as *V. vulnificus hemolysin* (*vvh*) (75).

RTX cytotoxin genes were restricted to *V. vulnificus*, with *rtxA*, *rtxB*, *rtxC*, and *rtxD* present in all *V. vulnificus* strains and absent in *V. navarrensis* and *V. cidicii*. This finding suggests a baseline of the virulence potential shared across the clade associated with the host but a specialized cytotoxic virulence limited to *V. vulnificus*. It is likely that *V. navarrensis* and *V. cidicii* utilize the core functions to evade the host immune response, but beyond that, there is no evidence of additional virulent traits. Our finding of a complete RTX gene absence in *V. navarrensis* and *V. cidicii* contrasts the results of Schwartz et al. (12), who reported RTX-positive *V. navarrensis* strains. This discrepancy likely stems from methodological differences and scale of analysis. Schwartz et al. used targeted BLASTN searches on a smaller subset of strains, while our study employed comprehensive screening across all available isolates using two independent approaches, ABRicate against the VFDB database and ROARY/SCOARY comparative analysis. Our broader sampling and dual method validation provide stronger evidence for the species-specific restriction of RTX toxins to *V. vulnificus*. Additionally, the use of stringent statistical criteria in our analysis reduces the likelihood of false-positive associations.

Moreover, *V. vulnificus* had additional virulence-associated genes, including those associated with iron scavenging (siderophores), hemolysin secretion, and host invasion/adherence. Compared to *V. cidicii* and *V. navarrensis*, which did not contain additional pathogenicity genes that were universally unique to each or either together, we hypothesize that *V. vulnificus* is an environmental and pathogenic specialist with a wider repertoire of virulence mechanisms than *V. cidicii* and *V. navarrensis*. Contrarily, *V. cidicii* and *V. navarrensis* appear to be environmental generalists with a smaller core virulence potential that is also shared with *V. vulnificus*.

Antimicrobial resistance (AMR) genes were observed across the *Vulnificus* clade, with many genes shared across all species, including resistance to tetracyclines, and with present *CRP*. This shared resistance pattern is typical of *Vibrio* species and has been documented in *V. cholerae*, *V. parahaemolyticus*, and *V. alginolyticus* (76). The presence of AMR indicates that there is likely vertical transfer of some conserved genes from a common ancestor not tied to the isolation location.

### One Health significance

These findings highlight the importance of a One Health approach to understanding the environmental reservoirs and transmission dynamics of *Vibrio* species. Genomic features core to the *Vulnificus* clade suggest that all three species are well adapted to respond to environmental change, which is essential for the transition from the environment to the host and the pathogenicity across kingdoms (77). The genomic features unique to *V. navarrensis* and *V. cidicii* provide evidence of anthropogenic-driven adaptation, which indicates a close proximity between these species in their aquatic environments and human populations. The phylogenetic clustering between clinical, environmental, and livestock isolates warrants future investigations into the transmission and pathogenesis of *V. navarrensis* and *V. cidicii*. By considering the interconnected systems of humans, animals, and the environment, we can offer deeper context for our findings.

To be pathogenic to humans and animals, environmental bacteria must be able to sense and respond rapidly to environmental change (77, 78). Genomic features conserved within the clade *Vulnificus* enable the rapid adaptation from aquatic to host environments. Among the core genome’s regulatory elements are important transcription factors, notably, sigma factors, including *rpoS*. The *rpoS* system is found across several pathogenic bacteria, where it functions to respond to environmental stress (nutrient-depletion, oxidative stress, osmotic shock, and pH changes) with adaptive transcriptional responses (79). In *V. vulnificus*, the *rpoS* system is important for motility and for responding to changes in osmolarity and pH (80). Its specific role in adaptive response in *V. navarrensis* and *V. cidicii* has not yet been investigated.

While iron is essential for many bacterial cellular processes, free iron is often limited in oceanic environments and in animal hosts. *Vibrio* species have adopted a range of mechanisms to acquire iron across these environments (57). For example, *V. vulnificus* employs several iron acquisition strategies, including siderophore synthesis to scavenge iron, heme receptors to bind to extracellular heme, and hemolysin synthesis to lyse host cells to release intracellular heme and iron (57). The genomic evidence in this study suggests that *V. navarrensis* and *V. cidicii* employ several of these same strategies. The clade’s core genome encodes the petrobactin-binding protein, YclQ, a siderophore most commonly attributed to *Bacillus* and *Alteromonas* species (81, 82). All three species encode the *hmuTUV* complex, which is involved in heme import, and *hutX*, which is involved in heme utilization (83).

While the synthesis of the vibriobactin siderophore (*vibB*) was restricted to *V. vulnificus*, all three species contained *viuB*, the gene encoding vibriobactin utilization. This indicates that, like many *Vibrio* species, *V. navarrensis* and *V. cidicii* have receptors for siderophores that they do not themselves synthesize (57). As proposed by reference 77, the range of iron acquisition strategies available to bacteria, such as *V. navarrensis* and *V. cidicii*, likely enables fitness across a range of environments and cross-kingdom hosts.

Transmission routes for *V. vulnificus* are well described. This ubiquitous marine organism colonizes many aquatic animals, including shellfish, without causing harm, but is pathogenic to some animals, such as eels (84). Humans can acquire *V. vulnificus* by consuming aquaculture products or through direct contact with contaminated waters. The transmission routes for *V. navarrensis* and *V. cidicii* remain poorly characterized; however, the genomic evidence presented in this study suggests waterborne transmission with potential for zoonotic spread. Additionally, a recent study has documented the presence of *V. navarrensis* in temperature-abused oysters (85), indicating a possible foodborne transmission route as well.

The anthropogenic-driven adaptation patterns observed in *V. navarrensis* and *V. cidicii* suggest that these species inhabit surface waters within close proximity to human populations. Most of the clinical isolates of *V. navarrensis* from the last decade in the USA cluster with *V. navarrensis* DA-9, an environmental isolate originating from seawater (23, 61). This clustering is consistent with aquatic environments serving as reservoirs of *V. navarrensis*, supporting zoonotic transmission through water or seafood exposure. Moreover, we found that two recent human clinical infections of *V. navarrensis* (*V. navarrensis* 2023V-1173 isolated in 2023 and *V. navarrensis* PNUSAV002826 isolated in 2021) clustered with livestock abortion cases when previously none had clustered in this way (11). These findings suggest that these infections could share a common transmission route likely related to aquatic exposures.

### Study limitations

Our study has some limitations inherent to a comparative genomic analysis of publicly available data. Assembly quality varies across the 76 genomes, with a mixture of complete and draft assemblies potentially affecting gene presence/absence detection. However, our conservative approach and focus on highly supported phylogenetic relationships mitigate these concerns. Additionally, as *V. cidicii* and *V. navarrensis* become more widely recognized and isolated, increased genomic sampling may reveal additional diversity. Because we have included only isolates characterized as members of the *Vulnificus* clade (1), it is possible that examination of the *Vulnificus* clade will need to be repeated as clade definitions are updated and as new isolates are described. For example, *Vibrio floridensis* has been identified as a novel species and is related to *V. vulnificus* (86); however, it is not currently included in the *Vibrionaceae* clades (1).

As *V. cidicii* and *V. navarrensis* become more easily characterized by sequencing applications and our understanding of environmental reservoirs, there will be greater genetic diversity that may alter our studies’ findings. Our findings on virulence are based on genomic content; functional characterization is needed to confirm phenotypic differences. Finally, because the isolation of *V. navarrensis* and *V. cidicii* is rare compared to *V. vulnificus* overall, we may not be capturing the full genomic diversity of these two species. This limited sampling constrains the strength of taxonomic proposals and highlights the need for continued genomic surveillance from geographically diverse locations to robustly assess species boundaries.

### Conclusions and future directions

This comprehensive genomic analysis of 76 genomes reveals that the *Vulnificus* clade represents a model system for understanding bacterial speciation in aquatic environments. While sharing core metabolic and survival functions, these three species have diverged in key areas: *V. vulnificus* has specialized virulence factors (RTX toxins) enabling severe human pathogenicity; *V. cidicii* has developed metabolic specializations (rhamnose catabolism) for specific environmental niches; and *V. navarrensis* occupies an intermediate position with broad environmental adaptability but reduced virulence potential. Both *V. cidicii* and *V. navarrensis* biotype *pommerensis* appear to have adaptations to survive environments with anthropogenic influence.

Our results indicate that *V. navarrensis* and *V. cidicii* are as closely related as the *V. navarrensis* biotype *pommerensis* is to *V. navarrensis*, raising important taxonomic questions that warrant continued investigation as additional genomes become available; however, the limited number of genomes currently available constrains definitive taxonomic conclusions at this time. Despite the absence of RTX toxins and presence of hemolysins, both *V. navarrensis* and *V. cidicii* continue to be isolated from clinical specimens, suggesting alternative pathogenesis mechanisms that warrant experimental investigation. Future work should include an experimental validation of virulence mechanisms and continued genomic surveillance as additional isolates become available to complement these genomic insights.

## Data Availability

The authors confirm that all supporting data, code, and protocols have been provided within the article. All accession numbers for genome assemblies used in this study can be found in Table 1. Supplemental materials and datafiles data files are available at OSF: https://doi.org/10.17605/OSF.IO/BH8Y2
